# Changing surface grafting density has an effect on the activity of immobilized xylanase towards natural polysaccharides

**DOI:** 10.1038/s41598-019-42206-w

**Published:** 2019-04-08

**Authors:** Cédric Y. Montanier, Mathieu Fanuel, Hélène Rogniaux, David Ropartz, Anne-Marie Di Guilmi, Antoine Bouchoux

**Affiliations:** 1 LISBP, Université de Toulouse, CNRS, INRA, INSA, Toulouse, France; 2 INRA, UR1268 Biopolymers Interactions Assemblies, F-44316 Nantes, France; 3 CEA, 18 route du panorama, 92265 Fontenay-aux-roses, France

## Abstract

Enzymes are involved in various types of biological processes. In many cases, they are part of multi-component machineries where enzymes are localized in close proximity to each-other. In such situations, it is still not clear whether inter-enzyme spacing actually plays a role or if the colocalization of complementary activities is sufficient to explain the efficiency of the system. Here, we focus on the effect of spatial proximity when identical enzymes are immobilized onto a surface. By using an innovative grafting procedure based on the use of two engineered protein fragments, Jo and In, we produce model systems in which enzymes are immobilized at surface densities that can be controlled precisely. The enzyme used is a xylanase that participates to the hydrolysis of plant cell wall polymers. By using a small chromogenic substrate, we first show that the intrinsic activity of the enzymes is fully preserved upon immobilization and does not depend on surface density. However, when using beechwood xylan, a naturally occurring polysaccharide, as substrate, we find that the enzymatic efficiency decreases by 10–60% with the density of grafting. This unexpected result is probably explained through steric hindrance effects at the nanoscale that hinder proper interaction between the enzymes and the polymer. A second effect of enzyme immobilization at high densities is the clear tendency for the system to release preferentially shorter oligosaccharides from beechwood xylan as compared to enzymes in solution.

## Introduction

Enzymes are proteins with specific catalytic activities, accepting only one type of substrate for catalysis, and that play a crucial role in cell metabolism. In some cases, instead of acting individually, they are part of complex organizations that involve several enzymes with distinct catalytic activities, as in the case of the ubiquitous glycolysis pathway for instance, in eukaryotes and even probably in prokaryotes^1,2^. Such enzymatic “cascades” fall into two major categories:

(i) enzymes are colocalized inside dedicated compartments like membrane-bound organelles (nucleus, ribosomes, Golgi apparatus, vacuoles, mitochondria) or microcompartments (carboxysome^3^, metabolosomes^4–7^).
(ii) enzymes are incorporated into one multifunctional enzyme complex (polyketide synthases^8^, tryptophan synthase^9^, fatty acid synthase^10^).

In both cases, the colocalization of the enzymatic activities has many potential beneficial effects on the bioprocess^11^. The reactants are concentrated relative to the total cell volume, thus driving reactions that can be unfavourable in dilute solutions^12,13^. The colocalization of enzymes may also reduce the feedback inhibition by reaction products^14^, decrease the loss of reactants and intermediates through diffusion and consumption of co-factors^15^ or prevent intermediate toxicity to the cell^16^. In addition, the proximity between the enzymes in compartments and their relative spatial organization in multienzyme complexes may facilitate the transfer of reaction intermediates directly from one enzyme to another, either by physical or electrostatic guidance^17–20^.

A fascinating example of a multienzyme complex (category (ii)) is the cellulosome described for the first time in the thermophilic bacterium *Clostridium thermocellum* in the early 80 s and that is involved in the degradation of the plant cell wall polysaccharides, primarily cellulose^21^. This enzymatic machinery consists of a non-catalytic scaffoldin protein decorated with cohesin modules, each of them being able to bind tightly to a dockerin module fused to a hydrolytic enzyme. The whole complex is attached to the bacterial outer membrane through a specific anchoring protein that contains a divergent dockerin^22^. Cellulosomes from different bacteria have been described with different levels of complexity, involving not only glycoside hydrolases (GHs) but also polysaccharide lyases and carboxyl esterases^23^. Also the questions of cellulosome composition and enzyme distribution along the scaffoldin have been extensively investigated, in many cases by using model and artificially designed mini-cellulosomes build from glycoside hydrolases mainly^24–28^. In these studies, it is clearly shown that a synergistic effect exists in the cellulosome, thus improving the reaction kinetics compared to the situation where the same enzymes are dispersed in solution^29^. A possible explanation is that the spatial proximity between enzymes in a cellulosome has the effect of concentring and localizing biocatalysts with complementary functions at specific sites on the lignocellulosic substrate.

The general question of the importance of spatial proximity between enzymes has been investigated by several groups in the past few years, but generic conclusions have not been forthcoming^30–37^. This is mainly because positioning enzymes onto surfaces at a controlled relative surface density and orientation remains challenging^38^. Recently, attempts have been made to immobilize GHs on solid supports in a cellulosome-like design^25,33,39–45^. However, in this configuration, the protein spatial proximity is not tuneable and the enzymatic activity is characterized only through the amount of product generated while the important issue regarding the effect of enzyme proximity on the chemical nature of the products (size, structure) has clearly been overlooked until now.

In the present work, we investigate the specific and generic question of how enzyme spatial proximity might influence the catalytic function. To do so, we develop an original system by grafting enzymes at various surface densities onto a surface, thus controlling the average relative distance between the biocatalysts. To keep our model as simple as possible, we use a single enzyme and therefore produce mono-component immobilized enzymatic systems that only differ in enzyme surface coverage. The model enzyme is the glycoside hydrolase (GH) *Neocallimastix patriciarum* endo-β-1,4-xylanase (*Np*Xyn11A) which belongs to family GH11^46^. This enzyme catalyses the hydrolysis of the β-1,4 glycosidic bonds in the backbone of xylan, a polymer of xylose residues. To orientate the enzyme and minimize interactions between the xylanase and the support, we use a biological conjugation method recently described that involves two small engineered proteins named Jo and In^47^. Jo and In present also the advantage of mimicking the size and shape of the cohesin/dockerin system. We show that this technique allows the immobilization of the enzyme without modifying its intrinsic activity, thus making the surface density (*i*.*e*. the average relative distance between enzymes) the only variable parameter to consider. The kinetic parameters and the product profiles generated by the enzyme are measured at various surface densities using simple (aryl-glycosides) and complex (polysaccharides) substrates. Our results reveal that the enzyme spatial proximity has a substantial impact on both the kinetics of the reaction and the nature of the products released.

## Results and Discussion

### Controlling the average distance between immobilized enzymes

**Figure 1:**
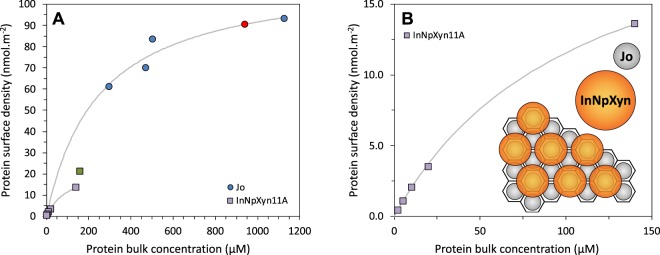
Quantity of immobilized proteins as a function of protein bulk concentration. (**A**) Jo immobilization on NHS-activated beads (circles) and In*Np*Xyn11A immobilization on beads pre-coated with ~90 nmol.m^−2^ of Jo (squares). The green square point in (**A**) is a result obtained with a distinct batch of beads and in conditions of precoating with Jo that are slightly different from the other points. (**B**) A magnified view of the data for In*Np*Xyn11A together with a schematic representation of the hypothetical arrangement of the immobilized enzymes at maximum occupancy. In all immobilizations, the concentration of beads is kept constant at ~15% volume fraction and the incubation lasts for 1 h. The lines are guides for the eye.

The enzyme immobilization protocol we developed is based on the use of two engineered protein fragments, Jo and In, that spontaneously and covalently attach to form a Jo-In complex^47^. Jo, which is immobilized onto a solid support, forms a covalent linkage with In itself fused to *Np*Xyn11A. In this way the xylanase is indirectly attached to the support. Commercially available porous paramagnetic beads of 10 µm diameter are used as support. The beads are both externally and internally lined with activated NHS carboxylic groups that react with primary amine of lysine residues of Jo to form stable covalent bonds (see Fig. S2). The quantity of Jo that is attached onto and into the beads is plotted in Fig. 1A as a function of the concentration of Jo in solution (circles). The coverage density is given in moles per surface area, where surface area refers to the actual available surface of the beads as measured through the BET method (see Material and Methods and SI for details).

The immobilized quantity of Jo increases with bulk concentration and reaches what appears to be a saturation plateau at high concentration. The increase in protein surface density with bulk concentration may be surprising at first as we are dealing with irreversible adsorption/immobilization that should result to the same surface coverage at equilibrium. This is, however, a common observation which can have multiple origins^48,49^: (i) an availability effect, i.e. at low concentration, the available quantity of Jo in the bulk is not sufficient to saturate the beads, (ii) a kinetic effect, i.e., the incubation time is not sufficient to reach saturation, thus resulting in less immobilized proteins at low protein concentration in the bulk when the immobilization kinetics is slower, (iii) a denaturing effect, i.e. at low bulk concentration, when the adsorption kinetics is slow, the immobilized proteins undergo structural changes that increase their footprint on the surface and limits further immobilization. This results in less coverage than at high bulk concentration when the adsorption kinetics is faster^50,51^.

At the highest concentration of Jo in the bulk (~1100 μM), additional experiments indicate that a 2 h incubation does not increase much the immobilized quantity of Jo (results not shown). This suggests that kinetic effects become negligible in that concentration range and that we are close to the ‘real’ saturation of the beads with Jo proteins. By simple extrapolation, we estimate the plateau value to ~114 nmol.m^−2^, which corresponds to a surface of 1/(*N*_A_ × 114 nmol.m^−2^) ≈ 14.5 nm^2^ per Jo molecule; where *N*_A_ is the Avogadro’s number. This value correlates well with the projected surface area of Jo protein estimated at 10–19 nm^2^ (see Fig. S2 in the SI for a 3D representation of the protein). This in turn suggests that the saturation level corresponds to the formation of a homogeneous and dense monolayer of Jo proteins on the available surface of the beads.

The second step of the enzyme immobilization procedure consists in incubating beads that are pre-coated with Jo with the enzyme In*Np*Xyn11A that contains In at its *N*-terminus. We used beads for which the Jo surface density is close to saturation (~90 nmol.m^−2^, red circle in Fig. 1A), with an estimated surface coverage of ~80%. The immobilized quantity of In*Np*Xyn11A is plotted in Fig. 1B as a function of the concentration of In*Np*Xyn11A in solution during incubation. The observed immobilization corresponds to the direct and specific interaction between Jo and In, as the non-specific immobilization of In*Np*Xyn11A on bare beads and of *Np*Xyn11A on Jo-coated beads is much lower in comparison (Table S1 in the SI). Also we have clear evidence that the immobilization is performed homogeneously on the accessible surface of the beads (see Fig. S3 in SI where confocal microscopy shows that the fluorescent protein GFP fused to In is immobilized homogeneously through the beads. As the sizes of GFP and *Np*Xyn11A are similar, 45.3 kDa and 41.5 KDa respectively, it is reasonable to assume that the xylanase is immobilized in the same manner).

As for Jo on bare beads, the quantity of immobilized enzymes increases with the bulk concentration. Again, this may be explained by a combination of the three effects described above for Jo. However in that case, denaturation effects are quite unlikely as the enzymes activity is fully preserved in all conditions of concentration and surface density (see next section). The estimated surface density at saturation is ~24 nmol.m^−2^, indicating that a maximum of ~27% (≈24/90) of the ‘Jo sites’ can be occupied by In*Np*Xyn11A molecules. As the monolayer of Jo is dense, we can reasonably assume that the Jo proteins are arranged on the surface in a way that is close to the ideal case of a hexagonal lattice. This results in an average center-to-center distance *d* =$\sqrt{\frac{2}{n\sqrt{3}}}$, with *n* the number surface density. We obtain *d* ≈ 4.6 nm for 90 nmol.m^−2^ of Jo; a value that is smaller than the estimated size of In*Np*Xyn11A (5–10 nm). As a consequence, two In*Np*Xyn11A molecules cannot occupy Jo sites that are immediately adjacent. Using simple geometrical considerations, we can demonstrate that the maximum occupancy in that case is 33%, i.e., one Jo site in three can be occupied by one In*Np*Xyn11A molecule in the lattice (cartoon in Fig. 1B). The experimental value of maximum occupancy is close to this estimation, indicating that specific immobilization *via* Jo-In is highly efficient. The slightly smaller value for the ‘real’ occupancy as compared to the theoretical one is probably due to some heterogeneities in the distribution of Jo proteins over the surface and/or to a small quantity of Jo sites that are no more able to interact with In-containing proteins (not accessible, wrong orientation, misfolding).

To summarize, our data indicate that we have an efficient method for irreversibly immobilizing a monolayer of enzymes onto the available surface of the beads. The surface density of enzymes can be varied by using different enzyme concentrations in bulk solution during immobilization. This allows the generation of beads in which the average distance between enzymes can be varied at will. In Table 1, we give estimations of the average center-to-center distances between In*Np*Xyn11A proteins that correspond to the densities of Fig. 1B. In addition to the distance values that are calculated assuming that the proteins are regularly spaced (hexagonal lattice assumption, more appropriate to dense packings), we provide another calculation based on the assumption that the proteins are randomly distributed on the surface (random Poisson process, more appropriate to dilute packings^52^). In the following sections, we provide a detailed analysis of the enzymatic activity of the beads that corresponds to these distance values.

**Table 1:** In*Np*Xyn11A surface density and average distance between In*Np*Xyn11A for the different beads produced in this study.

Beads identification number	In*Np*Xyn11A surface density (nmol.m^−2^)	Average center-to-center distance between adjacent In*Np*Xyn11A (nm)	
		Random Poisson process	Hexagonal lattice
0^*^	21.2	4.4	9.5
1	13.6	5.5	11.9
2	3.5	10.8	23.3
3	2.1	14.2	30.5
4	1.1	19.5	41.9
5	0.5	30.0	64.4

The distances *d* are calculated assuming that (a) the proteins are randomly distributed on the surface (Random Poisson process), with *d* = 0.5 *n*^1/2^ ^50^, or (b) the proteins are regularly spaced and arranged on a hexagonal lattice, i.e., *d* =$\sqrt{\frac{2}{n\sqrt{3}}}$. The exact average distances most probably lay between those two values. ^*^Results with beads 0 were obtained with a different batch of beads and in slightly different conditions of precoating with Jo as compared to beads 1–5 (green square point in Fig. 1A).

### Immobilization does not affect enzyme activity; only distance does

As a first verification, we checked if the simple addition of In at the *N*-terminus of *Np*Xyn11A, and the succeeding association of Jo with In*Np*Xyn11A has an effect on the activity of the enzyme, independently of the immobilization and therefore using free enzymes in solution. For that purpose the specific activities (SA) of *Np*Xyn11A, In*Np*Xyn11A, and Jo-In*Np*Xyn11A were evaluated using a short chromogenic oligosaccharide molecule as substrate (4-nitrophenyl-β-D-xylotrioside, *p*NP-X_3_)^46^. The substrate *p*NP-X_3_ can only be cleaved once by the enzyme. This releases X_3_ and 4-nitrophenol; the concentration of the last being followed by spectrophotometry to quantify the reaction. The SA is the molar number of cleavages per unit of time that perform the enzymes per milligram of enzymes. We find that neither the fusion with In nor the complexation with Jo affect the activity of the enzyme (SI, Table S3).

The activity of the immobilized enzymes was then assessed for the beads 1 to 5 listed in Table 2, still using *p*NP-X_3_ as the substrate. The measurements were performed using the same volume concentration of beads 1 to 5, each having different degrees of enzyme coverage (Table 2(A)), or using the same mass concentration of enzyme and therefore a varying volume concentration of beads with different degrees of enzyme coverage (Table 2(B)). For comparison (Table 2(C)), the specific activity of the free enzymes was also measured in a concentration range that corresponds to the enzyme concentrations of series (A). As expected, the SA of the free enzyme does not change with enzyme concentration and is constant at ∼2–2.5 μmol.min^−1^.mg^−1^. More importantly, we find that the SA of the enzymes immobilized on the beads, irrespective of the degree of coverage and bead concentration, is essentially identical to the SA of the free xylanase in solution. We conclude that the grafting procedure has no effect on the activity of the enzyme, meaning that the active site remains fully functional in all cases and that its efficiency is not affected by the average distance between the immobilized enzymes. In the SI, we further explore the effect of immobilization on the enzyme activity towards *p*NP-X_3_ by looking at the variation of SA with pH and temperature for both free and immobilized enzymes (Fig. S4). We find that some subtle differences exist between free and immobilized enzymes but that the general statement that the immobilized enzymes are intact and fully active is still perfectly true. Note that this conclusion implies that all the immobilized enzymes are immediately available to the substrate molecules during the enzymatic assays, which could be disputable as we are dealing with porous microbeads. In fact, we are very confident that this is the case as we estimate the time scale for substrate diffusion into the beads to be ≪1 minute (see the SI for an estimation of this diffusion time), which is very short compared to the time scale of the enzymatic assays (15 minutes during which the activity is fully stable).

**Table 2:** Specific activity of immobilized and free In*Np*Xyn11A against *p*NP-X_3_.

Specific activity SA (μmol.min^−1^.mg^−1^)			
Immobilized enzymes			Free enzymes
Beads	(A) at constant beads volume fraction = 0.3%	(B) at constant total concentration of In*Np*Xyn11A in solution = 4.47 mg/L	(C) at various In*Np*Xyn11A concentrations
1	2.12 (4.47)^a^	2.14 (0.3%)^b^	2.29 (4.47)^c^
2	2.88 (1.15)^a^	2.14 (0.7%)^b^	2.48 (1.15)^c^
3	2.52 (0.67)^a^	2.10 (1.3%)^b^	2.45 (0.67)^c^
4	2.56 (0.35)^a^	2.12 (2.7%)^b^	2.38 (0.35)^c^
5	2.19 (0.15)^a^	2.10 (5.9%)^b^	2.14 (0.15)^c^

The activity of immobilized In*Np*Xyn11A was assessed by either keeping constant the volume fraction of beads (0.3%, (A)) or keeping constant the total concentration of enzyme in solution (4.47 mg/L), (B)). The activity of free In*Np*Xyn11A was assessed at varying concentrations of enzymes corresponding to the concentrations of series (A). The values in brackets are: ^a^the equivalent concentration of enzyme that corresponds to 0.3% of beads volume fraction, as expressed in mg/L, ^b^the volume fraction of beads that corresponds to 4.47 mg/L of In*Np*Xyn11A, ^c^ the concentration of free In*Np*Xyn11A in solution, as expressed in mg/L.

We now look at the activities measured against a substrate that is not a short and synthetic oligosaccharide anymore but rather a ‘long’ polysaccharide that corresponds more directly to the reality of lignocellulose degradation in nature. In such a case, the xylanase can cleave the substrate in many potential positions. The degradation finishes when the substrate is cut into small elementary oligosaccharides that cannot be cleaved anymore. As a model of such substrate, we chose beechwood xylan, a polymer of β-(1,4) xylose units partially substituted with charged 4-O-methyl glucuronic acid units (Me-GlcA) and that is commercially available. In the solutions that we used, SEC-MALS analysis indicates that most of the xylan polymer (85% in mass, see SI for details, Fig. S5, Table S4) is present as chains of molecular weight 250–350 kDa. Such chains adopt random coil configurations that are separated or partially overlap with each other. Based on our SEC-MALS results and using reported data for similar polymers, we estimate the radius of gyrations R_g_ of these coils to 20–70 nm^53–55^. The other 15% in mass is present as high molecular weight objects (∼8 MDa) with a well-defined size (R_g_ ≈ 60 nm, see SI); presumably clusters of chains associated through non-covalent interactions. Quite importantly, we emphasize that the size properties of beechwood xylan are not sufficient to hinder or slow down the diffusion of the chains inside the porous beads in a way that impacts the activities that we report hereafter. A first direct indication of this is that all the measured activities were fully stable during the first 15–20 minutes of the enzymatic reaction, meaning that the same number of enzymes was involved during this time lapse. On the other hand, we know from confocal experiments performed with FITC-dextran of different sizes (70–500 kDa) that beechwood xylan most probably fully diffuses into the beads at times <10 minutes.

**Figure 2:**
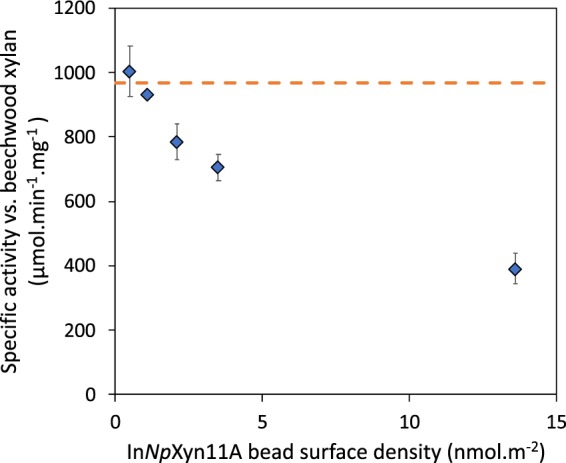
Specific activity of immobilized In*Np*Xyn11A against beechwood xylan as a function of the surface density of In*Np*Xyn11A on the beads. The horizontal dash line is the average specific activity of the free In*Np*Xyn11A. For the second point at low surface density, the error bar is too small to show given the symbol size. Experiments were performed in triplicate.

The specific activities measured with beads 1–5 against beechwood xylan are given in Table 3 and compared to the activity of the free enzyme. Here again, and as expected, the SA of the free enzyme does not change with its concentration as we are in excess of substrate. The SA measured with the beads are much more surprising as we clearly observe an increase of the activity with bead’s number. This indicates that the measured specific activity is all the more important that the density of In*Np*Xyn11A immobilized on the beads is low. To better illustrate this result, we plot in Fig. 2 the measured SA as a function of In*Np*Xyn11A density on the beads. At low surface density, the SA is similar to the free enzyme; which is another strong indication that the diffusion of xylan into the beads is rapid and has no effect on the measured enzymatic activities. When surface density increases, the SA activity decreases regularly until reaching less than half its initial value at the maximum In*Np*Xyn11A density.

**Table 3:** Specific activity of immobilized and free In*Np*Xyn11A against beechwood xylan.

Specific activity SA (μmol.min^−1^.mg^−1^)		
Immobilized enzymes		Free enzymes
Beads	(A) at 0.06% beads volume fraction	(B) at equivalent enzyme concentration^a^
1	391.2 ± 16.2 (0.99)^a^	919.0 ± 48.4 (0.99)^a^
2	705.4 ± 41.0 (0.25)^a^	971.2 ± 36.4 (0.25)^a^
3	784.4 ± 55.9 (0.14)^a^	934.0 ± 24.5 (0.14)^a^
4	932.9 ± 7.5 (0.08)^a^	922.1 ± 70.2 (0.08)^a^
5	1004.2 ± 79.4 (0.03)^a^	1092.5 ± 90.1 (0.03)^a^

(A) The activity of immobilized In*Np*Xyn11A was assessed at beads volume fraction 0.06%, (B) The activity of free In*Np*Xyn11A was assessed at equivalent enzyme concentration. ^a^The values in brackets are the total concentration of enzyme in the conditions of the tests, as expressed in mg/L. Experiments were performed in triplicate.

This result is further confirmed when looking at the Michaelis-Menten kinetic parameters determined for four of the 6 types of beads that we prepared (Table 4). The beads 5 display the lowest density of grafting and show kinetic parameters that are very similar to In*Np*Xyn11A in solution. The reduction in activity with increased enzyme density is then evidenced by both an increase in *K*_m_ and a decrease in *k*_cat_ for the other beads. This is particularly evident for beads 0 where immobilization of the xylanase at the highest density (21.2 nmol.m^−2^, ∼4.4–9.5 nm average distance between enzymes) results in a two-fold increase in *K*_m_ and a five-fold decrease in turn-over number *k*_cat_, resulting in a reduction of the catalytic efficiency of the immobilized In*Np*Xyn11A by a factor 10 compared to the free enzyme.

**Table 4:** Catalytic parameters of In*Np*Xyn11A in solution and immobilized onto beads using beechwood xylan as substrate.

	Free InNpXyn11A	Beads 0	Beads 2	Beads 4	Beads 5
Surface density (nmol.m^−2^)	—	21.2	3.5	1.1	0.5
*K*_m_ (mg.mL^−1^)	1.8 ± 0.7	3.6 ± 0.5	3.6 ± 0.5	2.6	2.1
*k*_cat_ (10^3^ min^−1^)	46.1 ± 8.8	8.9 ± 0.9	22.4 ± 2.7	43.5	55.1
*k*_cat_/*K*_m_ (10^3^ min^−1^.mg^−1^.mL)	25.6 ± 6.5	2.5 ± 0.07	6.2 ± 9.5	16.5	26.2

*Due to the high amount of beads 4 and 5 required, experiments could not be performed in triplicate. We provide two Michaelis-Menten graphs in the SI (Fig. S7) that correspond to these parameters: the free enzyme case and beads 4.

How to explain this decrease in activity against xylan with beads that are densely covered with enzymes? The explanation cannot be based on the intrinsic activity of the individual, immobilized enzymes as we know that all enzymes are active (Table 2). Also the fact that the activity of the free enzyme is recovered at low grafting densities clearly eliminates a potential limitation of the enzymatic reaction by the diffusion of the polymer inside the beads (an effect that would lead to always smaller activities compared to the free enzymes). So the explanation most likely relies on some steric effects relative to the size of the substrate and the average center-to-center distance between neighbouring enzymes: this distance being much smaller than when the enzymes are free in solution (Table S2). When this distance is small compared to the size of the polymer, a chain that interacts with one enzyme on the surface may occupy space in such a way that enzymes that are immediately adjacent are not available to other polymer chains or even cannot properly interact with other regions of the same polymer chain. As a consequence, these enzymes would become inefficient for a certain time lapse, which in turn would decrease the overall ‘activity’ of the system.

### Enzyme proximity and hydrolytic product profile

As shown previously, enzyme surface density has a direct effect on the overall activity of the system (i.e., number of cleavages per unit of time) against a natural polymer chain like beechwood xylan. An interesting question is whether such an effect has some repercussions on the nature of the products of the enzymatic reaction: do we observe any change in the profile (i.e., size distribution, nature) of the products when changing enzyme surface density and therefore the average distance between immobilized enzymes? To explore this question, we performed HPAEC-PAD and MALDI-ToF mass spectrometry (MS) on solutions of beechwood xylan that are progressively degraded by immobilized or free In*Np*Xyn11A.

**Figure 3:**
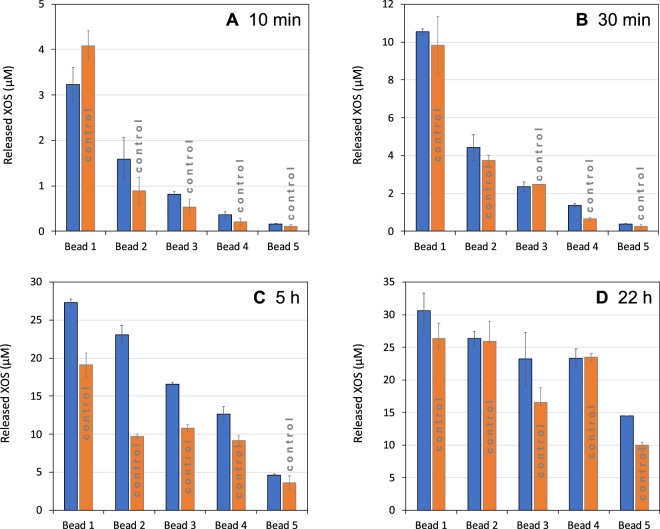
Concentration of xylooligosaccharides X_1_ to X_6_ released after (**A**) 10 min, (**B**) 30 min, (**C**) 5 h and (**D**) 22 h of hydrolysis of beechwood xylan with beads 1 to 5 (blue bars) and an equivalent concentration of free In *Np*Xyn11 A (control, orange bars). The activities of the immobilized enzymes were assessed at 0.13% beads volume fraction. The concentration of beechwood xylan was 2% w/v in all cases. Experiments were performed in triplicate.

HPAEC-PAD gives access to the quantity of xylo-oligosaccharides (XOS) with a degree of polymerization (DP) from 1 to 6 (X_1_ to X_6_) released by the action of the xylanase (Figs S6 and S7). In Fig. 3, we plot as blue bars the total amount of these XOS for beads 1 to 5 after short (10 min (A) and 30 min (B)) and longer periods of incubation (5 h (C) and 22 h (D)) in a 2% w/v beechwood xylan solution and using a same volume fraction of beads of 0.13% in each case. The red bars are the XOS quantities obtained with free enzymes at a concentration that corresponds to the total enzyme concentration in the bead’s experiments (named control experiments).

A first general observation is that the quantity of released XOS is always larger for beads 1 and then decreases with bead’s number; which is also the case with the control experiments. In fact this results is not a surprise as the experiments were performed at the same volume fraction of beads and consequently at variable enzyme concentrations: from 1.98 mg/L for beads 1 to 0.06 mg/L for beads 5. So quite simply, less XOS are released at a given time when less enzymes are used. Note that the difference in quantity of released XOS between the beads clearly becomes less marked as deconstruction occurs (see Fig. 3D in particular, after 22 h of hydrolysis). Again, this makes sense as the catalytic reaction becomes progressively limited by the quantity of substrate in the medium: the reaction tends to the production of short chains at a concentration that is only determined by the initial concentration of substrate^46,56,57^.

A second and much more interesting result of Fig. 3 is the difference in XOS quantity between each bead and its control experiment. In almost every case, and whatever the progress of the hydrolysis, the beads covered by enzymes release more XOS than if the same quantity of enzymes are free in solution. This is a particularly striking result as we know moreover that enzymes immobilized onto beads work slower than free enzymes when dealing with beechwood xylan (Table 3, Fig. 2): so even if cleavage events are fewer when enzymes are immobilized, the number of short oligosaccharides is greater than when enzymes are free in solution. This undoubtedly indicates that enzyme immobilization modifies the course of the deconstruction and favours the generation of short fragments of chains. Note that the results obtained with beads 1 at short time (Fig. 3A) seems to contradict this statement as more XOS are obtained with the control experiments. We believe this is related to the difference in SA between immobilized and free enzymes in that case. This difference is quite important at such a surface density (Table 3, Fig. 2), therefore even if immobilized enzymes preferentially generate short chains, the number of cleavages is not sufficient compared to the free enzymes that work randomly but are fast enough to generate more XOS after 10 minutes of deconstruction. At longer times, this effect is clearly supplanted by the effect of immobilization on the tendency of releasing short chains.

**Figure 4:**
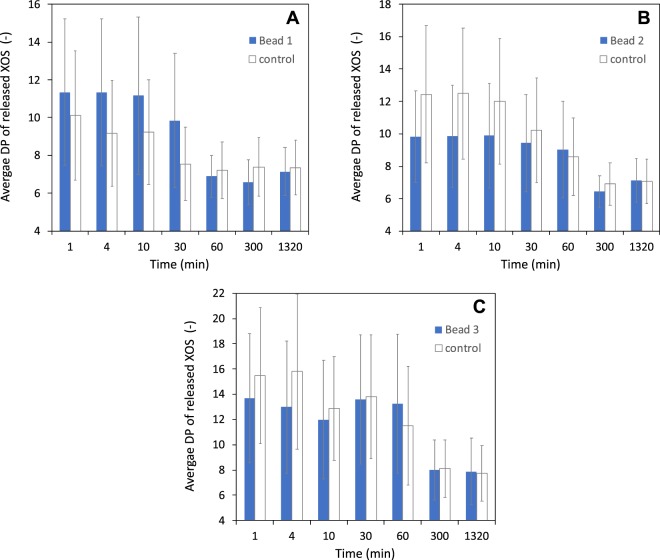
Average degree of polymerization of xylooligosaccharides (DP 3 to 30) released over the time detected by MALDI-TOF experiments (linear detector) with beads 1 (**A**), 2 (**B**), and 3 (**C**). The vertical bars are standard deviations and not error bars. They reflect the degree of size polydispersity of the released chains. The activities were assessed at 0.13% beads volume fraction. The concentration of beechwood xylan was 2% w/v in all cases. The square points are the results obtained from control experiments performed with free enzymes in solution and at equivalent concentration of the respective beads.

To complement these data, we performed MS experiments in order to identify XOS products on a wider range of DP (from 6 to ∼30). This technique makes it also possible to differentiate XOS that are substituted or not with MeGlcA units^58^ (Fig. S10 and Table S5). As a large diversity of unsubstituted and substituted oligosaccharides is generated, we simplified the results by reducing the species to their associated DP, independently of their substitution by MeGlcA. The contribution of a given DP to the mixture is then evaluated by summing all measured peak intensities attributed to this DP, no matter how substituted the XOS is. The average DP is obtained by normalizing this contribution to that of all DP. In Fig. 4, we plot the average DP for beads 1 to 3 and their control experiments as a function of time. The vertical bars that are associated to each value are the widths (standard deviations) of the DP distributions. They reflect the degree of size polydispersity of the released chains in each case.

The DP distributions obtained with beads 4 and 5 are very close to the DP distribution of the control experiments and are not presented here. At short times (t < 30 min), the MS results are generally consistent with the HPAEC-PAD results: beads 2 and 3 preferentially generate chains that are shorter than for free enzymes, whereas beads 1 generate chains that are slightly larger than for immobilized enzymes - probably for the same reason as the one we propose for explaining the HPAEC-PAD results in this particular case. At longer times (t > 30 min), there is no difference between immobilized and free enzymes, with, for all beads, an average DP that lies between 6–8. This is again consistent with the results of Fig. 3 and the fact that the catalytic reaction becomes limited by the quantity of substrate at those times and tends in all cases to the production of the same short chains. The narrowing of the DP distributions, as illustrated by the smaller standard deviation values obtained at t > 30 min as compared to short times, is another indication of this trend towards the production of chains of small and similar sizes. Note finally that we do observe certain differences, at short times, in the profile of MeGlcA substitutions between oligosaccharides released by free and beads-immobilized enzymes. We provide these results in Fig. S9 in the SI. We choose not to discuss them in the present work as the differences - even if interesting - are difficult to quantify and interpret without further experimental work.

To summarize, our HPAEC and MS analyses show unequivocally that, when in presence of a long polymer chain such as a xylan, enzymes immobilized on beads produce more short chains than enzymes that are simply dispersed in solution. This is an important result as it suggests that it is actually possible to control the characteristics of the products of the reaction through the immobilization of the enzymes at high surface densities. In a first analysis, it seems reasonable to relate this result to the average distance between adjacent enzymes on the beads, as we did for explaining the reduction in SA for enzymes that are densely packed onto beads (Fig. 2). As enzymes are close to each other when immobilized, there is more chance that they attack the same chain at positions that are also close to each other, which logically results in more short chains than when enzymes are free to move in solution and much more distant to each other (Table S2). One could also imagine that this mechanism would result in chains that are shorter when the average distance between immobilized enzymes is decreased. In fact, this is what we observe in MS for beads 2 and 3 (Fig. 4B,C), with an average DP of ∼10 for beads 2 (∼11–23 nm distance between enzymes) versus ∼14 for beads 3 (∼14–31 nm). However, we cannot conclude on that point as a rigorous examination of that question would require to have the full size distributions of the products for all beads and to compare these distributions at the same number of cleavage events rather than at the same hydrolysis time (as the kinetics depends on the surface coverage). This clearly represents a strong experimental challenge and is - in any case - beyond the scope of the present paper.

## Conclusion

In this paper, we investigated the effect of grafting the xylanase *Np*Xyn11A at various densities on porous paramagnetic beads both on the specific activity of the system and the size/quality of the products as compared to free enzymes in solution. Our results indicate that the specific activity of the individual grafted enzymes is not affected by the immobilization process that use the small protein fragments Jo and In to properly orientate the enzymes on the surface^47^. Also it is shown that the immobilization protocol can be tuned easily so that the surface coverage is controlled precisely; leading to average distances between adjacent enzymes that vary in the range 5–70 nm. When the enzymes are immobilized at high surface coverage and put in contact with beechwood xylan, a ∼250–350 kDa naturally-occurring polysaccharide, we find that (i) the overall activity of the system decreases, meaning that the enzymes work slower than when dispersed in solution, (ii) the system produces preferentially short oligosaccharides, still when compared to the products of the reaction when enzymes are free in solution. These are two important features that we believe are directly related to the close distances between neighbouring immobilized enzymes (10–30 nm) and are probably the consequence of some steric hindrance and geometrical effects at the nanoscale between the enzymes and the polymer chains. Further work is necessary to fully understand such effects, among which the experimentally challenging characterization of the size and nature (=substitution) distributions of all the products of the hydrolysis as a function of the number of cleavage events. In all cases, the present study could provide some important information for better understanding the mechanisms by which biological enzyme machineries work *in vivo*; in particular those systems where enzymes are packed at small distances between each other. Our findings may for instance partly justify why using a complex external membrane bound macromolecule like the cellulosome is an efficient strategy for lignocellulolytic microorganisms to generate rapidly small metabolizable oligosaccharides with minimum energy costs for the cell^59^. Additionally, we believed that our results are clearly of industrial interest and could participate to the optimization of processes involving immobilized enzymes.

## Materials and Methods

### Jo/In and *Np*Xyn11A properties

The recently published Bio Molecular Welding toolbox^47^ was used to immobilize enzymes on solid support. This toolbox provides two small engineered protein fragments named Jo and In which are able to create an autocatalytic intramolecular isopeptidic bond. As solid support, commercial paramagnetic beads of 10 µm diameter (PureProteome™ NHS FlexiBind, Merckmillipore) were used. These beads display carboxylate activated residues by *N*-hydroxysuccinimide (NHS) ester groups in order to react with primary amino groups, mainly from lysine residues exposed at the surface of proteins^60^. In order to immobilize the enzyme of interest, Jo was used to functionalize the support while the enzyme was expressed in fusion with the complementary In and later associated to the beads. Thus, enzyme was covalently bound to the solid support *via* Jo-In association. (see below for details).

Plasmids pBMW1 and pBMW2 coding respectively for an His-tagged In and Jo protein and plasmid pADG16-InsfGFPop coding for an His-tagged superfolded variant of GFP were a gift from Anne-Marie Di Guilmi and Thierry Vernet^47^. The xylanase 11 A from *Neocallimastix patriciarum* (*Np*Xyn11A^46^) was chosen as the model enzyme and was sub-cloned as the fusion protein In*Np*Xyn11A in the plasmid pET28-In*Np*Xyn11A (see details in SI). Neither Jo nor In proteins display a tryptophan residue in their amino acid sequence^47^, making the determination of the protein concentration by measuring the absorbance at 280 nm not accurate enough. Thus, a tryptophan residue was introduced by point mutation in the MCS1 of both pBMW1 and pBMW2 (see details in SI). Additional tryptophan did not affected the Jo-In complex formation (see Fig. S1). For convenience, resulting proteins were still named Jo and In respectively. Proteins were expressed and purified as described in SI section 1.

### Protein immobilization on paramagnetic beads

Commercially available porous paramagnetic beads of 10 µm diameter were used as solid support (PureProteome™ NHS FlexiBind, Merckmillipore). The specific surface area is 37.8 m^2^ per gram of dried beads, as determined by N_2_ adsorption-desorption (see SI for details). Based on the specifications given by Merckmillipore for these beads (ligand density >17 μmoles NHS per mL settled beads), it is reasonable to consider that the NHS groups are present all over the inner and outer surface of the beads. Morever, the surface densities obtained after Jo immobilization, together with the results of complementary experiments performed with fluorescent proteins (Fig. S3) indicate that the totality of the surface of the beads is accessible and available for protein immobilization. The first step of grafting consisted in the immobilization of Jo. Every steps were performed at 21 °C under constant agitation at 1,000 rpm (ThermoMixer® C, Eppendorf) in 2 mL centrifuge tube. Aliquot of 50 µL of homogeneously re-suspended solution containing 20% (v/v) beads was washed with 500 µL of ice-cold 1 mM HCl. Beads were then incubated for 1 h with 60 µL of Jo (about 450 µM) in 50 mM sodium phosphate, pH 7. Beads were washed 3 times 20 s with 500 µL of 50 mM sodium phosphate, 150 mM ethanolamine, pH 7 and incubated with an additional 500 µL for 1 h in order to quench residual NHS. The beads were eventually washed 2 times with 500 µL of 50 mM sodium phosphate, 250 mM NaCl, 1% triton X-100, pH 7 and 2 times with 500 µL of 50 mM sodium phosphate, pH 7. The amount of immobilized Jo was determined as described in SI (protein grafting measurements). The second step of grafting was done by mixing beads displaying Jo with 80 µL of adjustable concentrations of protein of interest (In, In*Np*Xyn11A, InsfGFP) in 50 mM sodium phosphate buffer, pH 7. Beads were washed 2 times with 500 µL of 50 mM sodium phosphate, 250 mM NaCl, 1% triton X-100, pH 7, 1 time with 500 µL of 100 mM MES, 500 mM NaCl pH 5, 1 times with 500 µL 100 mM Tris/HCl, 500 mM NaCl, pH 8 and 1 time with 500 µL of 50 mM sodium phosphate, pH 7. Beads were stored in 100 µL of 50 mM sodium phosphate, pH 7 at 4 °C (10%, v/v). The amount of immobilized protein (In, In*Np*Xyn11A, InsfGFP) was determined as described in SI section 1.

### Enzymatic activity

#### Enzyme assays

Dinitrosalicylic acid (DNSA) assay^61^ was performed to estimate reducing sugar when beechwood xylan (sigma) was the substrate. Substrate concentration was set to 2% w/v, and all the xylan solutions were prepared by mixing the dry powder with water during at least 30 mins at 90 °C. The enzymatic reactions were performed at 37 °C under constant agitation at 1,400 rpm (ThermoMixer® C, Eppendorf) in 2 mL centrifuge tube. Enzymatic assay were assessed as previously described^46^ in 12 mM sodium citrate, 50 mM sodium phosphate buffer pH 6 supplemented with 1 mg/mL of BSA. At regular time, aliquot of 100 µL was mixed to 100 µL of DNSA and incubated 10 min at 95 °C. After cooling down on ice, 1 mL of deionized water was added. If required, magnetic beads were trapped using a magnetic stand (PureProteome™ Magnetic Stand, 8-well, Merckmillipore). Samples of 300 µL were displayed on a 96 wells microplate and absorbance at 540 nm was measured using a microplate spectrophotometer (Eon Microplate Spectrophotometer, Biotek). Aliquots of D-xylose were prepared from 0 to 2 mg/mL as standard curve.

The kinetic parameters using 4-nitrophenyl-β-D-xylotrioside as substrate were determined by measuring absorbance at 401 nm of the released 4-nitrophenolate (ε = 12,578 M^−1^.cm^−1^). For the free xylanase (Table 2(C)), 450 µL of 50 mM sodium phosphate, 1 mg/mL of BSA, pH 7 containing 5 mM of 4-nitrophenyl-β-D-xylotrioside was preheated at 37 °C for 5 min in quartz cuvettes with a chamber volume of 500 µL (cuvettes Hellma Analytics), using a spectrophotometer Cary 100 Bio (Agilent Technology). The reaction was then initiated by the addition of 50 µL of the xylanase. As for the In*Np*Xyn11A coated-beads (Table 2(A,B)), the solutions were prepared by carefully sampling the required volumes of bead solutions using calibrated pipette and under constant agitation of the bead tubes. Due to bead sedimentation, enzyme assay with immobilized In*Np*Xyn11A was performed using a ThermoMixer® C (Eppendorf) under constant agitation at 1,400 rpm. Aliquot of 50 µL was withdraw and instantly mixed with 200 µL of 1 M Na_2_CO_3_. Beads were removed using magnetic stand and supernatant pipetted down to a 96 wells microplate. Absorbance at 401 nm (ε = 22,209 M^−1^.cm^−1^) was measured using a microplate spectrophotometer (Eon Microplate Spectrophotometer, Biotek). Specific activities were determined using beechwood xylan or 4-nitrophenyl-β-D-xylotrioside at concentrations 10 and 2.5 times over the *K*_m_ values, respectively (1.8 mg.mL^−1^ and 2.1 mM^44^). The kinetic parameters were calculated using non-linear regression in SigmaPlot (Systat Software, San Jose, CA).

#### HPAEC-PAD

Quantification of short xylooligosaccharides released over the time from beechwood xylan by free and immobilized In*Np*Xyn11A were determined using aliquots (200 µL) removed at regular time intervals and heated at 95 °C for 10 min to terminate the reaction. Each sample was centrifuged at 20,000 × g for 5 min and quantified by HPAEC-PAD using a Dionex ICS 3000 dual chromatography system. Xylooligosaccharides were separated on a Carbo-Pac PA-100 guard and analytical column PA-100 (2 × 50 mm and 2 × 250 mm). Separation of oligosaccharides was achieved by isocratic elution with 100 mM NaOH at a flow rate of 1 mL/min from 0 to 10 min, a gradient of 0 to 75 mM sodium acetate in 100 mM NaOH from 10 min to 25 min, and isocratic elution with 500 mM sodium acetate in 100 mM NaOH from 25 min to 35 min, then re-equilibrate the column with 100 mM NaOH for another 10 min. Calibration was achieved using xylose and xylooligosaccharides (X_2_, X_3_, X_4_, X_5_ and X_6_) at concentrations from 5 µg/mL to 40 µg/mL. All experiments were performed in triplicate, and reported values are the means of three experiments.

#### Mass Spectrometry (MS)

To simultaneously monitor xylooligosaccharides substituted or not by residues of methyl glucuronic acid, the samples collected from HPAEC-PAD were subsequently analyzed by matrix-assisted laser desorption/ionization (MALDI)-time-of-flight (ToF) MS. For the measurements, an ionic preparation of 2,5-dihydroxybenzoic acid (DHB) and N,N-dimethylaniline (DMA) was used as the MALDI matrix, as described in^62^. An equimolar mixture of 2,5-dihydroxybenzoic acid (DHB) and N,N-dimethylaniline (DMA) DMA (DHB 100 mg.mL^−1^, in H_2_O/acetonitrile/ DMA (1:1:0.02)) forming the matrix was mixed with the samples in a 1:1 ratio (v/v), and the mixture (1 μl) was deposited on a polished steel MALDI target plate. MALDI measurements were performed on an Autoflex Speed MALDI‐TOF/TOF spectrometer (Bruker Daltonics, Bremen, Germany) equipped with a Smartbeam laser (355 nm, 1000 Hz) and controlled using the Flex Control 3.0 software package. The mass spectrometer was operated with positive polarity in both reflectron and linear modes to cover the widest range of *m/z* possible. Spectra were acquired in the range of 400–2500 *m/z* and in the range of 1000–10000 *m/z* for the reflectron mode and for the linear mode, respectively.

## Supplementary information

Changing surface grafting density has an effect on the activity of immobilized xylanase towards natural polysaccharides
