# Supplementary material for: Changing surface grafting density has an effect on the activity of immobilized xylanase towards natural polysaccharides
Source: Sci Rep. 2019 Apr 8;9:5763. doi: 10.1038/s41598-019-42206-w (PMC6453946; doi:10.1038/s41598-019-42206-w)
Supplement: Supplementary file 1 — Changing surface grafting density has an effect on the activity of immobilized xylanase towards natural polysaccharides [file 41598_2019_42206_MOESM1_ESM.pdf]

SUPPORTING INFORMATION FOR

Changing surface grafting density has an effect on the activity of immobilized xylanase towards natural polysaccharides

Cédric Y. Montanier[1], Mathieu Fanuel[2], David Ropartz[2], Hélène Rogniaux[2], Anne-Marie Di Guilmi[3], Antoine Bouchoux [1]

[1] LISBP, Université de Toulouse, CNRS, INRA, INSA, Toulouse, France

[2] INRA, UR1268 Biopolymers Interactions Assemblies, F-44316 Nantes, France

[3] CEA, 18 route du panorama, 92265 Fontenay-aux-roses, France

This supplementary Information contains 4 sections:

1. Enzyme production and purification (Figure S1)
2. Enzyme immobilization onto paramagnetic beads (Figures S2-S3, Tables S1-S2)
3. Enzymatic activity (Figures S4-S7, Tables S3-S4)
4. Nature of the products released (Figures S8-S11, Table S5)

1. Enzyme production and purification

Plasmids pBMW1 and pBMW2

To minimize the effect of introducing an aromatic residue to the protein sequence of Jo and In, a glutamine residue next to the His tag at the *N*-terminus of plasmid pBMW1 and pBMW2 was replaced by a tryptophan using a QuickChange Lightning Site-Directed Mutagenesis Kit (Agilent technology) and the following primers: 5' TTT CTT TTC AGT CGG ATC CCA GCT GTG GTG ATG ATG GTG A 3', 5' TCA CCA TCA TCA CCA CAG CTG GGA TCC GAC TGA AAA GAA A 3' AND 5' CTG GTC AGA CGG ATC CCA GCT GTG GTG ATG ATG G 3', 5' CCA TCA TCA CCA CAG CTG GGA TCC GTC TGA CCA G 3'. Both resulting mutants Jo^{Trp} and In^{Trp} were expressed and purified as described and their ability to form a covalent bond was assessed and compared to Jo and In wild type using SDS-PAGE imaging analysis. As displayed in Fig. S1, protein complex formation was not affected by the presence of a tryptophan, complete reaction being achieved in 60 min.

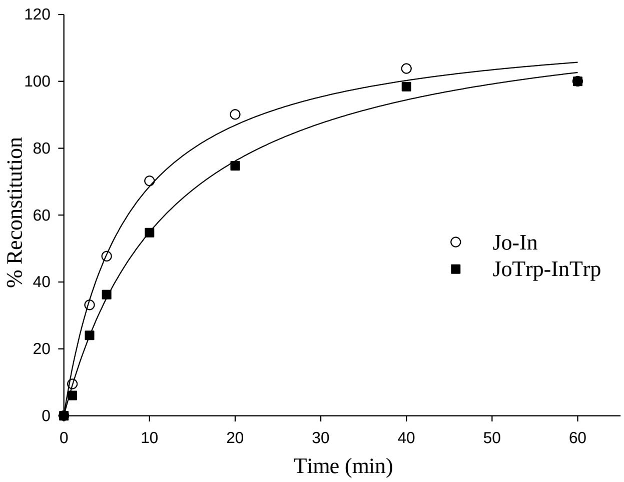

Figure S1. Comparison of complex formation of Jo-In and mutants Jo^{Trp}-In^{Trp}.

Plasmid pET28-In*Np*Xyn11A

The region of *in* gene was amplified by PCR using the following primers: 5’ GGA GAT ATA CCA TGG GCA GCA GCC ATC ACC ATC ATC 3’ and 5’ GAT TGA CCA TGC TAG CGC TGC CGC GCG GCA CCA GGT CGA CGT TAT CAC GAA TCT TAG GAA TCG GG 3’, which

introduce an BamHI and an NheI site. The PCR product was introduced by homologous recombination (In-Fusion® HD cloning kit, Clonetech) into a pET28b linearized vector using NcoI and NheI to produce pET28-In. The region of *xyn11A* gene was amplified by PCR using the following primers: 5' TCG TGA TAA CGT CGA CAA GTT TAC TGT CGG TAA TGG ACA AAA CCA AC 3' and 5' GTG CGG CCG CAA GCT TGT TAG GCT GGA CTA GAA CCC TTT GGA G 3', which introduce an SalI and an HindIII site. The PCR product was introduced by homologous recombination (In-Fusion® HD cloning kit, Clonetech) into a pET28-In linearized vector using SalI and HindIII to produce pET28-In*Np*Xyn11A. The resulting gene codes for the *N*-terminal His-tagged fusion protein In*Np*Xyn11A with a short linker of three amino acid between the *N*-terminal In and *C*-terminal *Np*Xyn11A domains.

Protein purification

The strain, BL21 (DE3) of *Escherichia coli* harboring plasmids of interest was cultured to mid-exponential phase (A_{600nm} 0.6) in Luria-Bertani broth at 37 °C. Recombinant enzyme expression was induced by the addition of isopropyl-β-D-thiogalactopyranoside to a final concentration of 0.5 mM at 37 °C for 4 h. Cells were harvested by centrifugation at 5,000 × g for 10 min, re-suspended in 10 ml of 50 mM sodium phosphate buffer, pH 7 containing 300 mM NaCl 20 mM imidazole and a protease inhibitor cocktail (cOmplete Protease Inhibitor EDTA-free, Roche) and lysed by sonication on ice for 1 min. The lysate was clarified by centrifugation (30 min at 74,000 × g at 4 °C). Proteins were purified by immobilized metal ion affinity chromatography (IMAC) using Talon resin (Clontech) and elution in 50 mM sodium phosphate buffer, pH 7 containing 300 mM NaCl and 150 mM imidazole. The eluted proteins were desalted using a PD-10 desalting columns (GE Healthcare Life Sciences) or extensively dialyzed against 50 mM sodium phosphate buffer, pH 7. A final round of purification was carried out using a XK16 HiLoad 16/600 75 prep grade gel filtration column (GE Healthcare Life Sciences) connected to an Äkta pure system. Typically, 1 ml of protein was loaded onto the column at 1 ml/min using a 1 ml static loop and finally eluted from the column using 50 mM sodium phosphate buffer, pH 7 containing 150 mM NaCl. Purified proteins were adjudged homogenous by SDS-PAGE. Protein concentrations were determined by measuring absorbance at 280 nm and applying the Lambert-Beer equation. Theoretical molar extinction coefficients were calculated using ProtParam online software[1].

2. Enzyme immobilization onto paramagnetic beads

Beads specific surface using BET

The nitrogen adsorption–desorption isotherms were collected at 77 K using an adsorption analyzer BELSORP-Mini II (BEL-Japan). Aliquot of 340 µl of beads (20 % v/v) were washed three times with water and dried under vacuum for 16 h at 21 °C, resulting into 0.0523 g of material. From the N_2 isotherm, the specific surface area (SBET) was determined by the BET method in $0.10 \leqslant P/P0 \leqslant 0.30$ domain. The errors associated with adsorption–desorption analyses were estimated to be 4% for the specific surface.

Protein immobilization on paramagnetic beads

From the crystal structure of the complex[2], Jo displays four solvent exposed lysine (K162, K168, K173, K213) and one additional lysine is involved in the intra molecular isopeptide bond (K191), whereas In presents eight solvent exposed lysine (K590, K591, K648, K651, K673, K690, K703, K717). Preliminary data clearly highlighted a higher efficiency of specific immobilization when Jo is firstly immobilized on the beads (data not shown). This behavior could be explained regarding the distribution of the solvent exposed lysine on the surface of Jo which are at the opposite of the catalytic residue Lys_{191}, instead of more exhibited around the catalytic Asn_{695} in the case of In (Fig. S2). Such lysine distribution may promote appropriate orientation of Jo and enable access to the catalytic Asn_{695} of In.

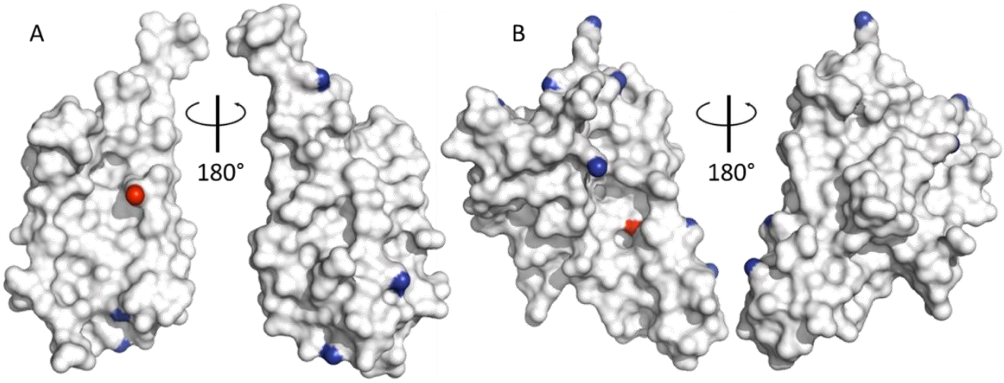

Figure S2. Surface representation of Jo (A) and In (B), twisted from 180°. Amine groups of solvent exposed lysine are in blue. In red, side chain Lys_{191} of Jo and side chain Asn_{695} of In involved in the amide bond formation of JoIn. The Jo protein fits in a cuboid of ~3.2×3.2×5.9 nm. A reasonable

estimation of its projected surface area is between 3.2×3.2 ≈ 10 nm^2 and 3.2×5.9 ≈ 19 nm^2. Images obtained using pymol software from pdb 2ww8, (The PyMOL Molecular Graphics System, Version 2.0 Schrödinger, LLC).

Table S1. Amount of In*Np*Xyn11A or *Np*Xyn11A immobilized to different pre-coated beads with Jo, In or buffer.

Coated Bead	Enzyme	Amount of enzyme per µl of beads (µg)
Jo	In*Np*Xyn11A	0.96
Jo	*Np*Xyn11A	0.10
In	In*Np*Xyn11A	0.26
In	*Np*Xyn11A	0.11
Buffer	In*Np*Xyn11A	0.39
Buffer	*Np*Xyn11A	0.20

Table S2. Comparative distance of In*Np*Xyn11A immobilized on Jo coated paramagnetic beads and equivalent amount of free In*Np*Xyn11A in solution. *Result obtained with a different batch of beads and in slightly different conditions of precoating with Jo as compared to beads 1-5. [a] The average center-to-center distances between adjacent immobilized enzymes are calculated based on the assumption of a random Poisson process (minimum value) or an hexagonal lattice arrangement (maximum value). See the article for details. [b] The reaction volume is 1.5 ml. [c] According to Erickson[3].

Average center-to-center distance between adjacent In*Np*Xyn11A (nm)		
Beads identification number	Immobilized enzyme[a]	Equivalent amount of enzyme in solution[b,c]
0*	4.4-9.5	348.5
1	5.5-11.9	409.6
2	10.8-23.3	645.9
3	14.2-30.5	774.9
4	19.5-41.9	976.4
5	30.0-64.4	1269.8

Protein grafting measurements

The amount of protein (Jo, In, In*Np*Xyn11A and InsfGFP) adsorbed on the paramagnetic beads was determined by measuring the depletion of the supernatant solution after equilibration of the sample. Each immobilization was assessed using 50 µl of beads (20 %, v/v) in 50 mM sodium phosphate buffer (pH 7). The samples were equilibrated for 1 h at 21 °C at 1,000 rpm in closed centrifuge tube using a thermomixer (ThermoMixer® C, Eppendorf). After equilibration, unbounded proteins were isolated from magnetic beads using a magnetic stand (PureProteome™ Magnetic Stand, 8-well, Merckmillipore). Immobilization of Jo to NHS beads released NHS in the medium displaying high absorbance at 260 nm (data not shown). Thus, the amount of protein in the supernatant containing Jo was determined by SDS-PAGE (Any kD™ Mini-PROTEAN® TGX Stain-Free™ Protein Gels, Biorad). The gel images were acquired using a Gel Doc EZ system (Biorad). The intensities of the colored bands corresponding to Jo (Mw = 10659.5 Da, ε = 15930 $M^{-1}.cm^{-1}$) were quantified using Image Lab™ Software (Biorad) and compared to the intensity of the initial loading. Protein concentration in the supernatant solution containing In (Mw = 16507.1 Da, ε = 12950 $M^{-1}.cm^{-1}$) was determined by UV-spectrometry at 280 nm using 2 µl of solution (Nanodrop 2000, Thermo Scientific). The amount of In*Np*Xyn11A immobilized to the beads (Mw = 41544.8 Da, ε = 69330 $M^{-1}.cm^{-1}$) was determined using the specific activity of the enzyme. Final concentration of 5 mM of 4-nitrophenyl-β-D-xylotrioside in 50 mM sodium phosphate, 1 mg/ml BSA, pH 7 at 37 °C (spectrophotometer Cary 100 Bio, Agilent Technology). Protein concentration in the supernatant solution containing InsfGFP (Mw = 45374.6 Da, ε = 27975 $M^{-1}.cm^{-1}$) was determined by fluorescence (Excitation wavelength = 395 nm, Emission wavelength = 504 nm, Infinite 200 Pro, Tecan).

Surface coverage homogeneity using confocal microscopy and InsfGFP

Beads were analyzed with a Confocal Laser Scanning Microscope (TCS SP2-SE, Leica, Germany) using a 63X oil immersion objective lens (numerical aperture 1.40, PL APO). Fluorescence was excited with the 488 nm ray line of the argon laser and recorded in one of the confocal channels in the 500 to 550 nm emission range. Stacking of images and fluorescence measurement were performed with Image J software[4]. Paramagnetic beads (10 % v/v) carrying InsfGFP ranging from 278 to 5 nmole / µl of beads (beads 0 to 6) were diluted 20 times in 50 mM sodium phosphate, pH 7 and 10 µl was spread on microscope glass slice with cover slip.

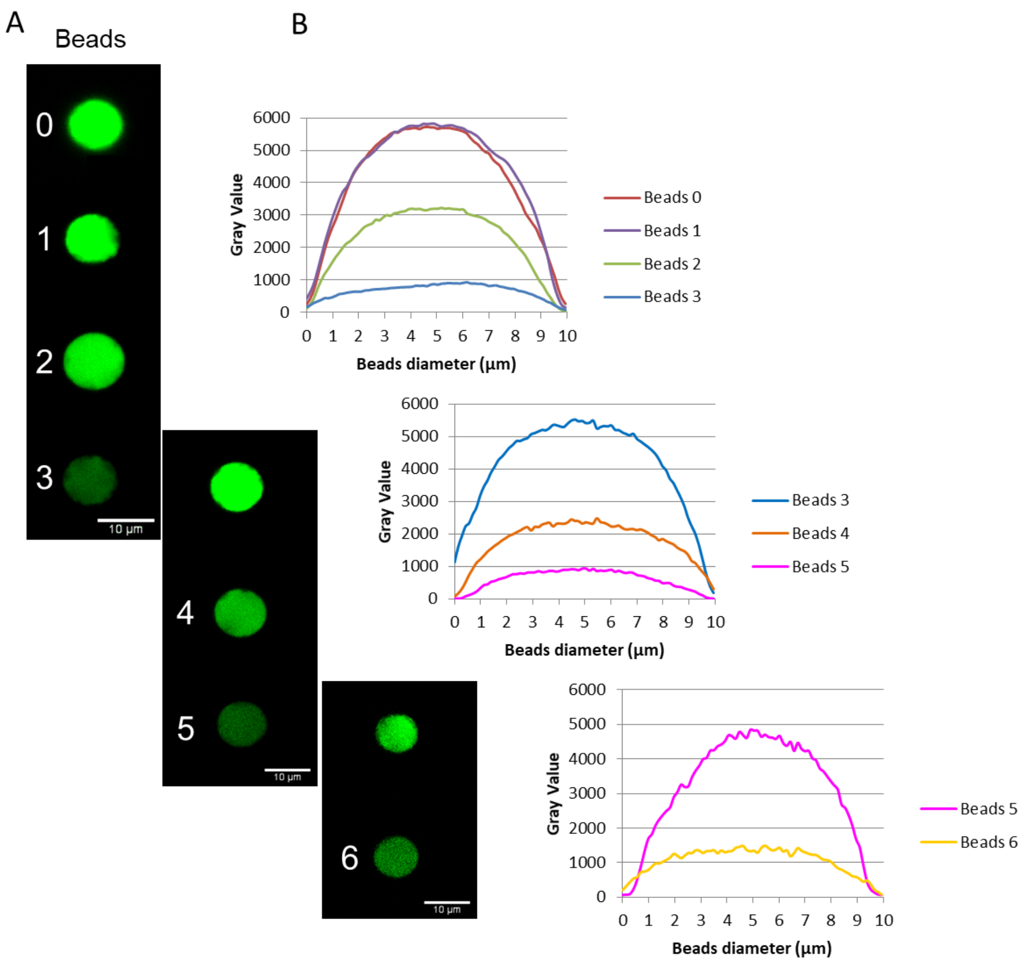

Figure S3. Decreasing amount of InsfGFP grafted on Jo coated paramagnetic beads. (A) Confocal fluorescence microscopy image of paramagnetic beads 0 to 6 on witch decreasing amount of InsfGFP was immobilized *via* JoIn association. The image corresponds to the middle of the bead. (B) Stacks of confocal images obtained for the different beads. Total fluorescence was measured along the diameter of the beads. In order to compare beads one to another and because of the large magnitude in fluorescence (factor 50), beads with the lowest intensity for one set comparison was used a second time as the highest intensity for the second set of comparison.

3. Enzymatic activity

Effect of the In and Jo on the intrinsic activity of *Np*Xyn11A

Table S3. Specific activity of *Np*Xyn11A and derivate. Activity was assessed against 5 mM of 4-nitrophenyl-β- D-xylotrioside in 50 mM sodium phosphate buffer pH 7 supplemented with 1 mg/ml of BSA, at 37 °C. IU = $\mu mol.min^{-1}$.

Enzyme	SA ($IU.mg^{-1}$)
*Np*Xyn11A	3.75 ± 0.35
In*Np*Xyn11A	3.35 ± 0.3
Jo-In*Np*Xyn11A	3.16 ± 0.23

An estimation of *p*NP-X_3 diffusion time into the beads

*p*NP-X_3 has a molecular weight of 535.45 $g.mol^{-1}$ and is about 21×6×6 Å in size. The diffusion coefficient D of a Brownian particle of diameter d can be estimated using the Stokes-Einstein expression $D = k_B T/6\pi\mu(d/2)$ with k_B the Boltzmann constant, T the temperature, and μ the viscosity of the medium (taken as water). At room temperature, this gives a lower limit of D = 2.04 × 10^{-10} $m^2.s^{-1}$ for pNP-X_3 if we use its largest dimension 21 Å as an equivalent diameter d. In addition, we know that the 3D root mean square displacement of a Brownian particle with a diffusion coefficient D in a time t is given by $<r^2>^{1/2} = (6Dt)^{1/2}$.[5] This means that pNP-X_3 diffuses a distance r = 10 microns in a time t $\approx r^2/6D$ = 0.08 s. If we assume that the diffusion into the beads is not hindered by porosity or obstruction effects, this means that pNP-X_3 takes less than one second to fully enter the beads. If, on the other hand, we hypothesize that the diffusion coefficient into the beads is 1% of D because of some strong obstruction effects (this percentage is very exaggerated, see the review of Masaro et al. on the diffusion of tracers in polymer solutions or gels[6]), the time for diffusing into one bead is still 8 s; which is also less than 1 minute as stated in the article.

Optimal pH and temperature

The effect of pH on free or immobilized *Np*Xyn11A was determined using 5 mM of 4-nitrophenyl-β-D-xylotrioside *p*NP-X_3. Aliquot of 50 µl was withdraw and instantly mixed with 200 µl of 1 M Na_2CO_3. Beads were removed using magnetic stand. The system was composed of 50 mM citrate buffer for pH 4.0 to 6.0, 50 mM phosphate buffer for pH 6.0 to pH8.0, 20 mM bicine buffer for pH 8.0 to 9.0 and 20 mM glycine/NaOH buffer for pH 9.0 to 10.0. The apparent optimal temperature of free or immobilized *Np*Xyn11A was determined over the range from 25 °C to 50 °C in 12 mM sodium citrate, 50 mM sodium phosphate buffer, 1 mg/ml of BSA, pH6. Aliquot of 50 µl was withdraw and instantly mixed with 200 µl of 1 M Na_2CO_3. Beads were removed using magnetic stand. Absorbance at 401 nm (ε = 22,209 $M^{-1}.cm^{-1}$) was measured using a microplate spectrophotometer (Eon Microplate Spectrophotometer, Biotek). Reactions were performed in triplicates.

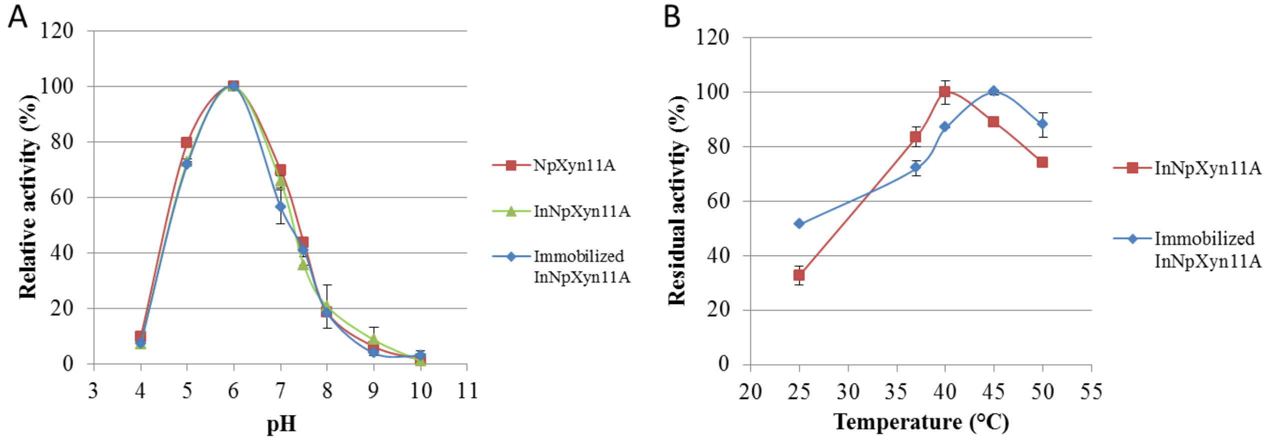

Figure S4. (A) Optimum pH of free and immobilized *Np*Xyn11A. Enzyme activity was measured at 37 °C in a pH range between 4 and 10 using 5 mM of 4-nitrophenyl-β-xylotrioside. (B) Optimum temperature of free and immobilized *Np*Xyn11A was measured at pH 6 using 5 mM of 4-nitrophenyl-β-xylotrioside. Activity was expressed as percent residual activity. Although the published optimum pH of *Np*Xyn11A was referred to be 7.5, assays with Oat spelt xylan were performed at pH 6.5 (data not shown[7]). Differences in substrate or presence of a His-tag may explained such pH optimum variation.

Molecular weight of beechwood xylan polymer

SEC-MALS (size exclusion chromatography coupled with multi-angle light scattering) was used to characterize the beechwood xylan sample used in our study. SEC was performed using three columns (1 Shodex SB-807 HQ and 2 Agilent PL aquagel-OH Mixed-M), and elution in NaCl 0.2 M and NaN3 0.1g/L at a flow rate of 1 mL/min. The beechwood samples was diluted so that the total concentration of xylan was 1 g/L. The detection was performed through the recording of both the refractive index (Shodex RI-101 detector, T = 35 °C) and light scattered at 18 angles (MALS Wyatt Dawn Heleos, λ_{laser} = 658 nm). The chromatogram obtained is given in Fig. S5

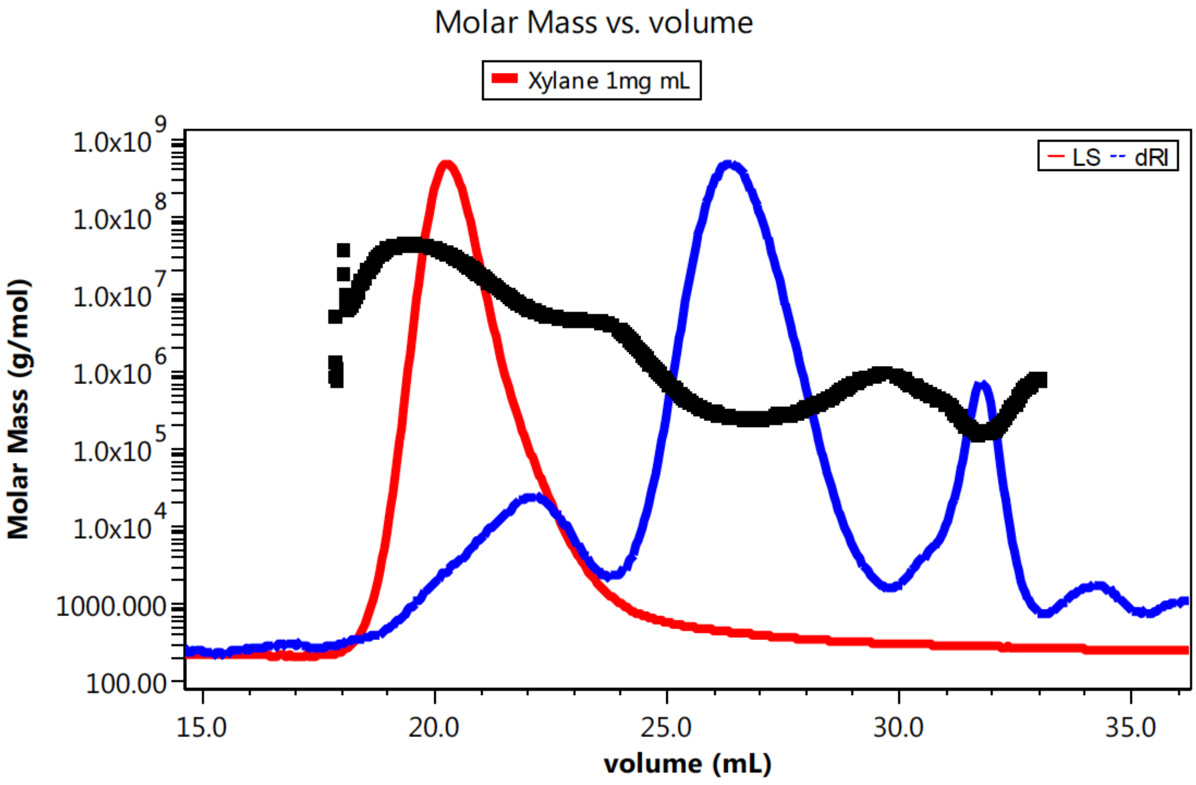

Figure S5. SEC-MALS analysis of beechwood xylan. LS: light scattering detector. dRI: refractive index detector.

The dRI chromatogram present three distinct peaks: peak 1 centered at 22 mL of elution, peak 2 at 27 mL, peak 3 at 32 mL. Peak 1 only is well resolved by LS; it represents 15 % of the total mass of beechwood xylan; while peaks 2 and 3 represent 85 % of beechwood xylan. The average molecular weight of each fraction is reported in the table below (Table S4).

Table S4. SEC-MALS analysis of beechwood xylan. Number (M_n) and weight (M_w) average molecular weight, polydispersity index (Đ =Mw/Mn), radius of gyration (R_g).

Beechwood Xylan	Mn	Mw	Đ	Rg
Peak 1	7,808,000	14,260,000	1.8	59
Peak 2	335,200	469,500	1.4	63
Peak 3	252,000	331,200	1.3	70

Most of the xylan polymer is therefore present as chains of molecular weight 250-350 kDa (number average molecular weight). The other part is present as much larger objects (7-14 MDa); presumably clusters of chains associated through non-covalent interactions.

The R_g values are estimated from the intensities scattered at different angles following Zimm's theory. The average R_g calculated for peak 1 is fully reliable. The R_g values for peaks 2 and 3 are less trustworthy and probably overestimated as the LS signal is still polluted by large objects that correspond to peak 1 at these elution times.

Surface accessibility using confocal microscopy and FITC-Dextran

Paramagnetic beads (10 % v/v) carrying In*Np*Xyn11A at 40 nmole / µl of beads were diluted 10 times in 50 mM sodium phosphate, pH 7 and 10 µl were mixed with 200 µl of 2 % Dextran FITC displaying different molecular weight (FITC-Dextran 500,000 $g.mol^{-1}$, 150,000 $g.mol^{-1}$, 70,000 $g.mol^{-1}$, 20,000 $g.mol^{-1}$) and incubated 10 min or 1 h at 37 °C under constant agitation (1,000 rpm, ThermoMixer® C, Eppendorf). After incubation, beads were washed 3 times in 200 µl using Dextran with similar molecular weight (Dextran 503,000 $g.mol^{-1}$, 70,000 $g.mol^{-1}$, 6,000 $g.mol^{-1}$, 1,500 $g.mol^{-1}$). Finally, beads were recovered in 40 µl of corresponding Dextran and 10 µl were spread on microscope slide with a cover slip. Stacking of images and fluorescence measurement were performed with Image J software[4].

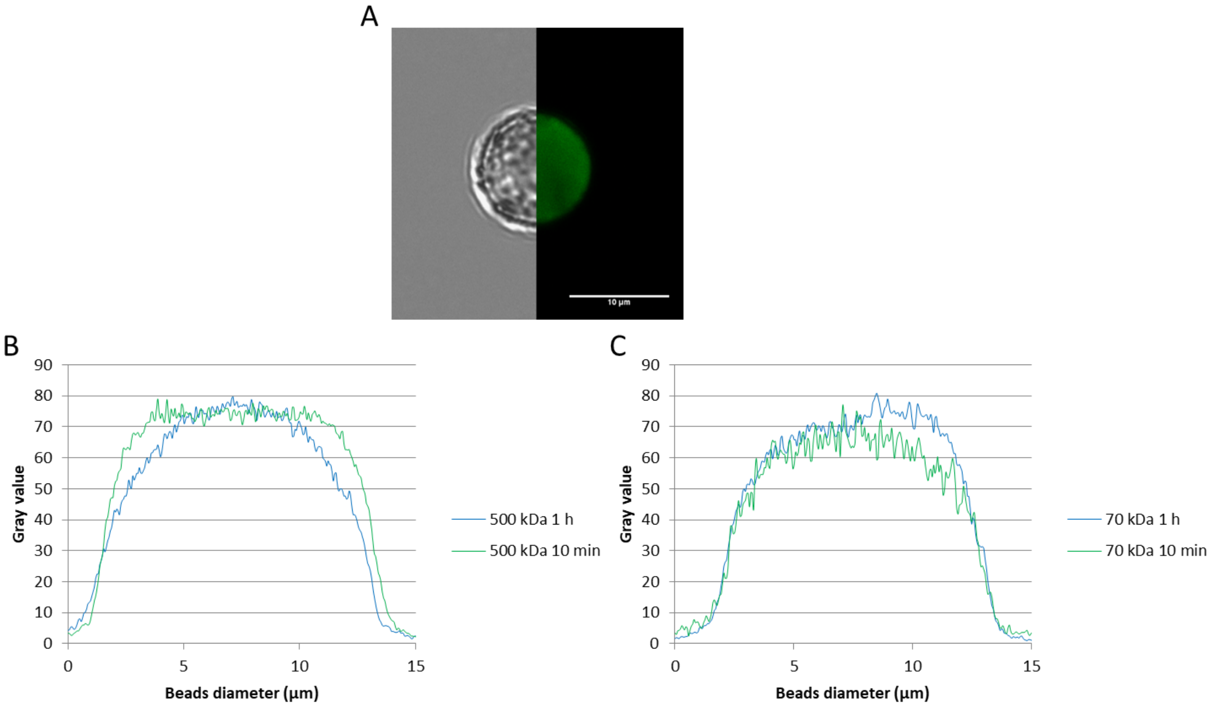

Figure S6. (A) Confocal fluorescence microscopy image of paramagnetic beads 1 incubated 10 min with dextran FITC 500 kDa. (B) Stacks of confocal images obtained for beads 1 incubated 10 min and 1 h with dextran FITC 500 kDa and (C) with dextran FITC 70 kDa. The bell curve reflects the spherical shape of the beads of diameter around 10 µm.

Catalytic parameters of In*Np*Xyn11A

In the next figure we provide two examples of the Michaelis-Menten curves from which the catalytic parameters of In*Np*Xyn11A towards beechwood xylan were determined (Table 4 in the article).

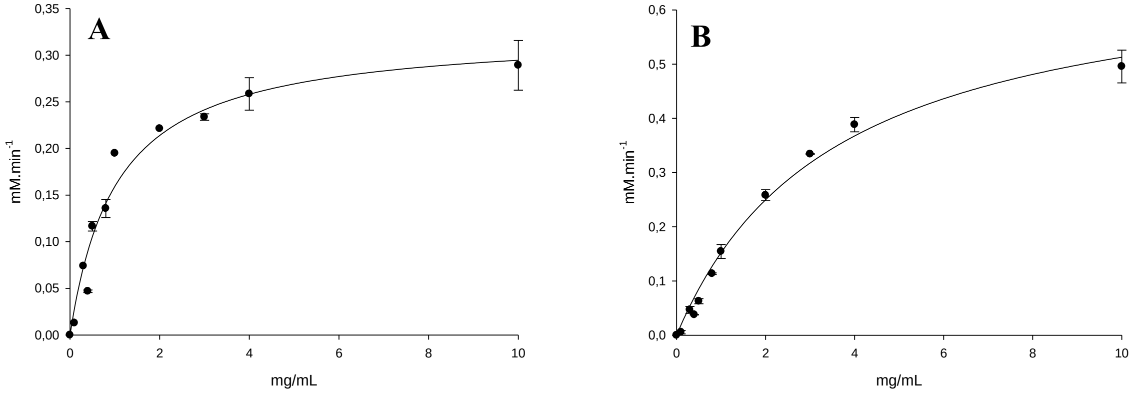

Figure S7. Michaelis-Menten curves for (A) free In*Np*Xyn11A and (B) immobilized In*Np*Xyn11A proteins (beads 4) using beechwood xylan as substrate. Enzyme activity was measured at 37 °C, in 12 mM sodium citrate, 50 mM sodium phosphate buffer pH 6 supplemented with 1 mg/ml of BSA under constant agitation. Beechwood xylan was used as substrate, in a range from 0.1 to 10 mg/mL.

4. Nature of the products released

HPAEC-PAD

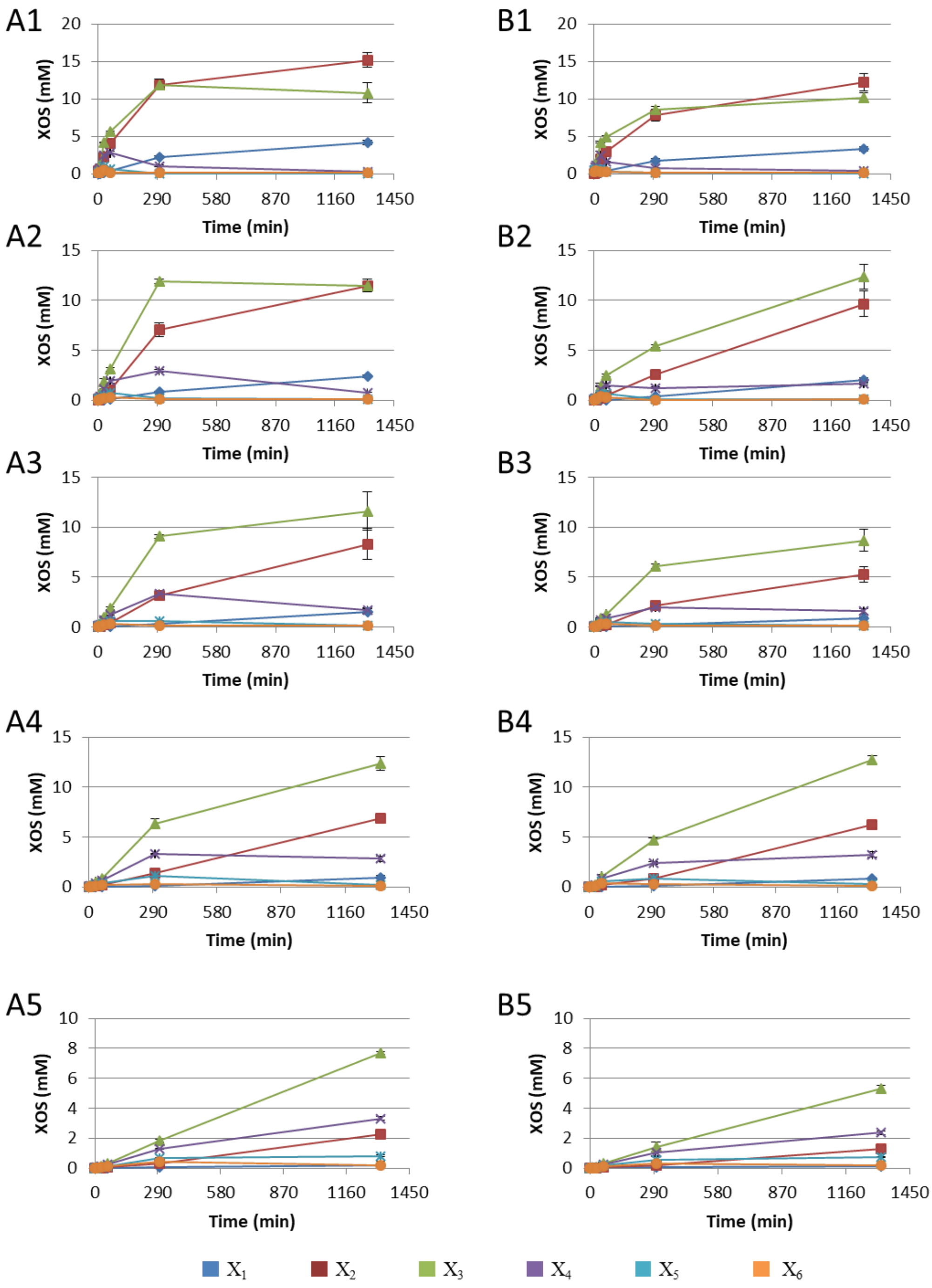

Figure S8. Production of xylooligosaccharides X_1 to X_6 from beechwood xylan by (A1 to A5) immobilized In*Np*Xyn11A referring to beads 1, 2, 3, 4 and 5 and (B1 to B5) equivalent amount of free In*Np*Xyn11A.

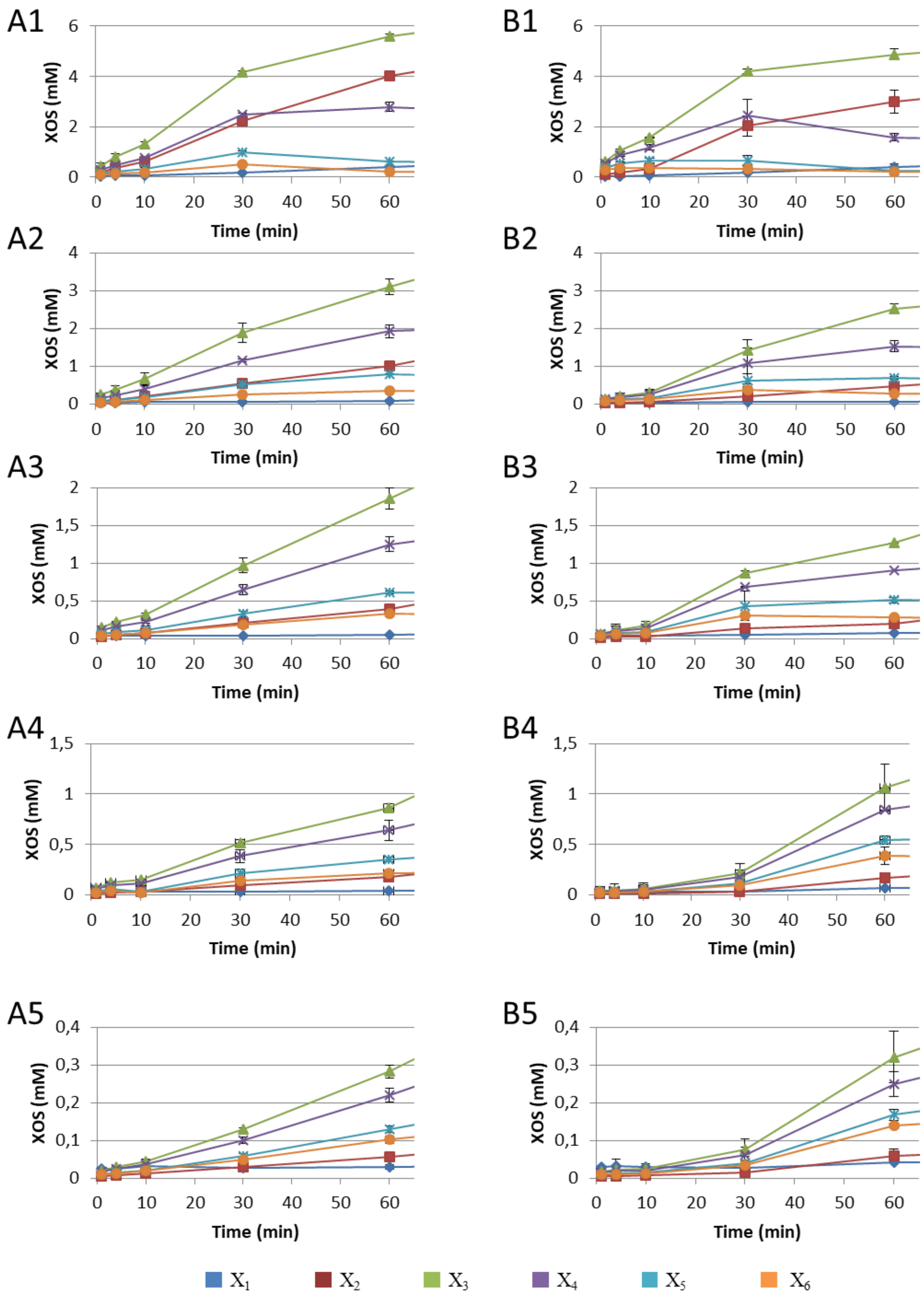

Figure S9. Closer view of the 60 first minutes of the kinetics displayed in Fig. S6. Production of xylooligosaccharides X_1 to X_6 from beechwood xylan by (A1 to A5) immobilized In*Np*Xyn11A referring to beads 1, 2, 3 and 5 and (B1 to B5) equivalent amount of free In*Np*Xyn11A. Immobilized xylanase released significant amount of XOS from the very beginning of the reaction in a constant manner whereas XOS produced by the enzymes in solution are released after a 10 to 30 min.

Mass Spectrometry (MS)

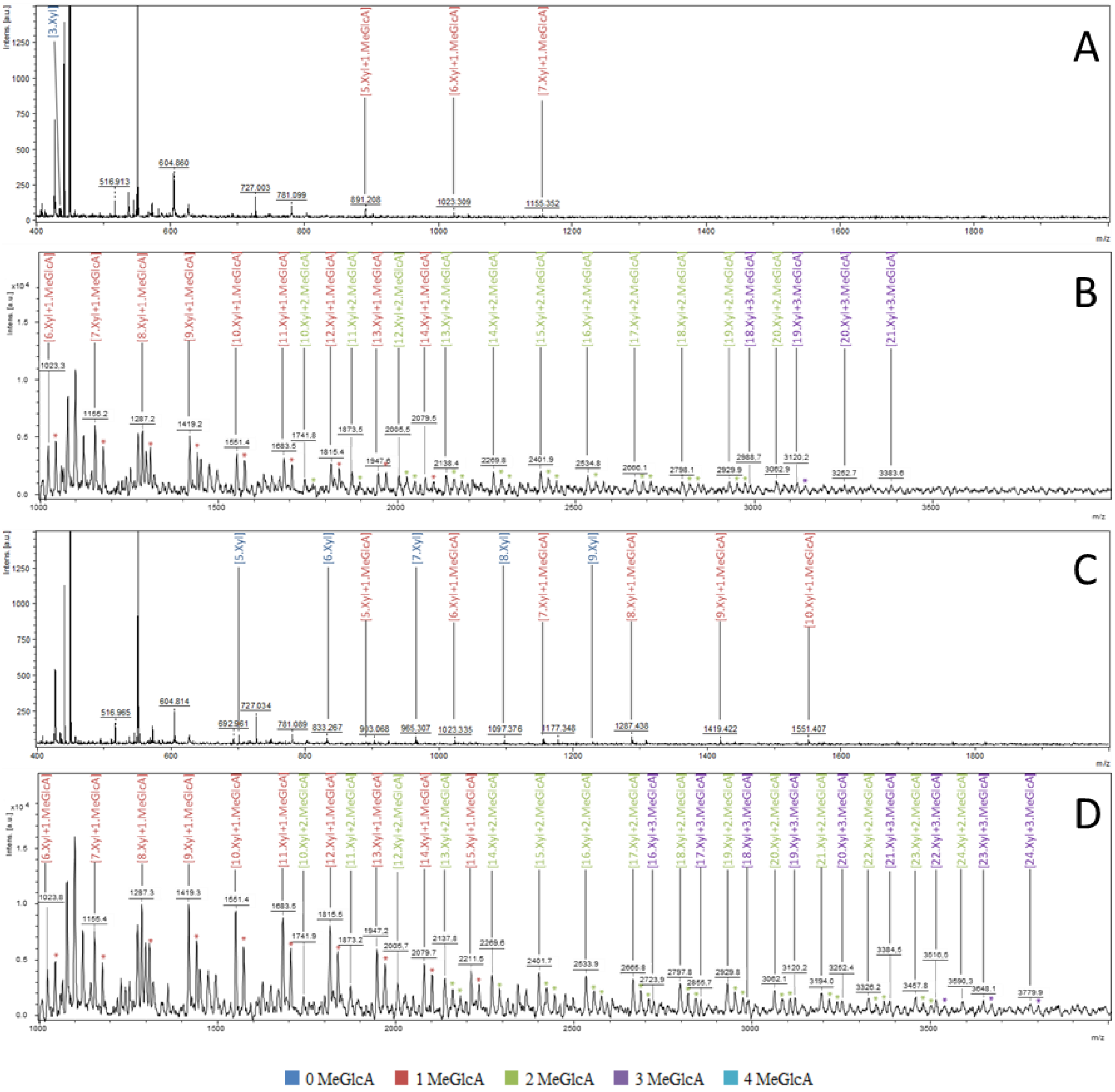

Figure S10. MALDI MS analysis of the xylooligosaccharides substituted with none, 1, 2 , 3 or 4 MeGlcA units released by the action of immobilized In*Np*Xyn11A on beads 2 ((A) in reflectron mode, (B) in linear mode) or equivalent amount of In*Np*Xyn11A in solution ((C) in reflectron mode, (D) in linear mode) after one minute of digestion. (* indicate a sodium adduct(s) on an annotated specie).

Table S5. Range of xylooligomers and corresponding m/z according to the type of the detector. NS refers to the number of MeGlA substitutions.

		Oligomers			
		Smallest		Largests	
Mode	NS	structure	m/z	structure	m/z
reflectron	0	X_3	437.1	X_{10}	1361.4
	1	$MeGlcAX_4$	759.2	$MeGlcAX_{12}$	1818.5
	2	$MeGlc_2AX_6$	1213.4	$MeGlc_2AX_9$	1609.5
linear	0	X_9	1229.3	X_{10}	1361.4
	1	$MeGlcAX_6$	1023.2	$MeGlcAX_{17}$	2475.8
	2	$MeGlc_2AX_6$	1213.4	$MeGlc_2AX_{29}$	4250.5
	3	$MeGlc_3AX_{10}$	1821.6	$MeGlc_3AX_{30}$	4595.1
	4	$MeGlc_4AX_{12}$	2385.3	$MeGlc_4AX_{24}$	3992.5

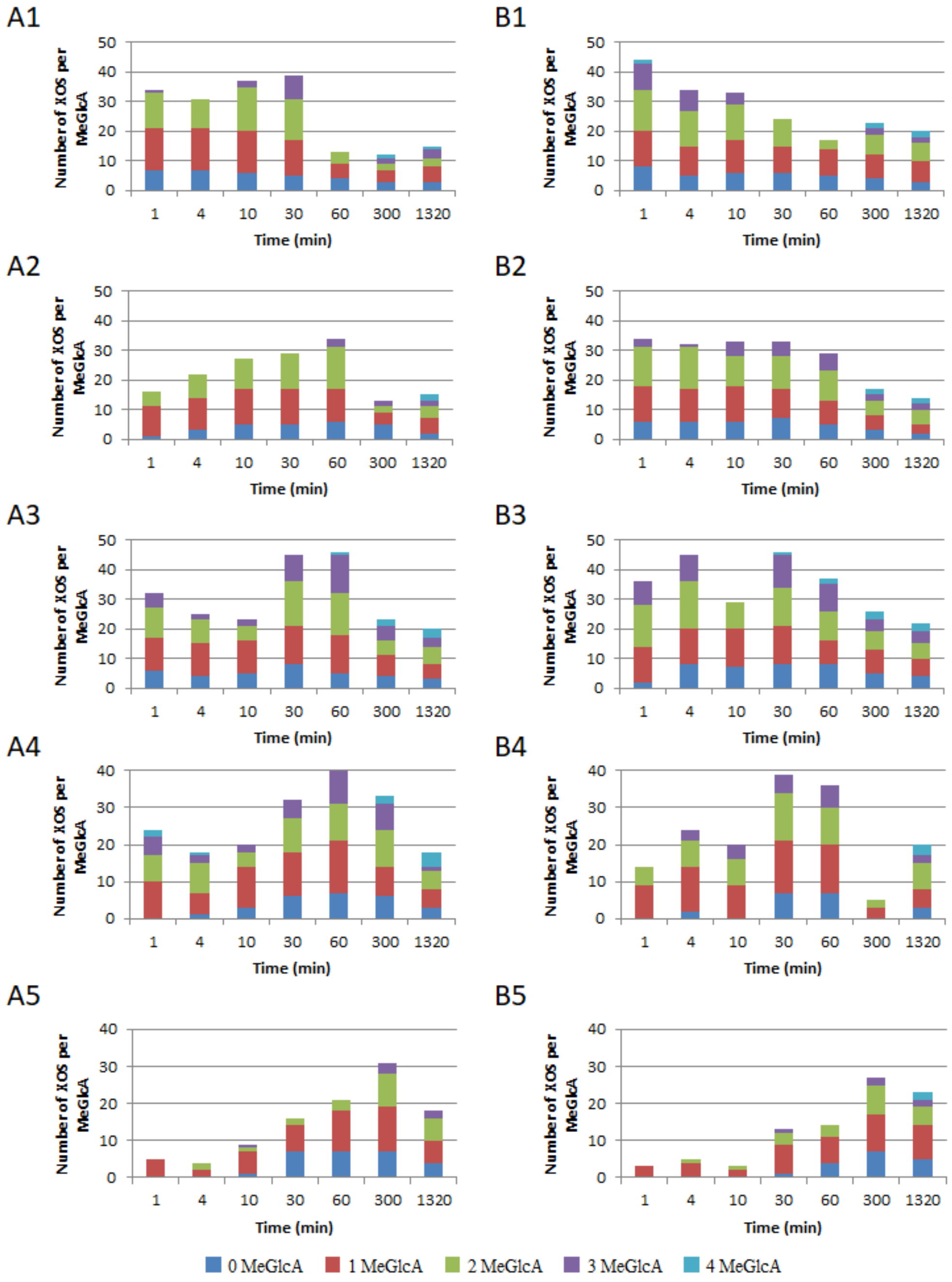

Figure S11. Number of the different xylooligomers substituted with none, 1, 2, 3 or 4 MeGlcA units. The different products were generated by the action of immobilized In*Np*Xyn11A (A) or In*Np*Xyn11A in solution (B) on beechwood xylan. A1 to A5 referred to beads 1 to 5 respectively. B1 to B5 referred as the corresponding equivalent amount of free enzymes.

References

1. Gasteiger, E. *et al.* Protein Identification and Analysis Tools on the ExPASy Server. in *The Proteomics Protocols Handbook* 571–607 (Humana Press, 2005). doi:10.1385/1-59259-890-0:571
2. Izoré, T. *et al.* Structural Basis of Host Cell Recognition by the Pilus Adhesin from Streptococcus pneumoniae. *Structure* **18,** 106–115 (2010).
3. Erickson, H. P. Size and Shape of Protein Molecules at the Nanometer Level Determined by Sedimentation, Gel Filtration, and Electron Microscopy. *Biol. Proced. Online* **11,** 32–51 (2009).
4. Schneider, C. A., Rasband, W. S. & Eliceiri, K. W. NIH Image to ImageJ: 25 years of image analysis. *Nat. Methods* **9,** 671–675 (2012).
5. Berg, H. C. *Random Walks in Biology, New and Expanded Edition*. (1993).
6. Masaro, L. & Zhu, X. . Physical models of diffusion for polymer solutions, gels and solids. *Prog. Polym. Sci.* **24,** 731–775 (1999).
7. Vardakou, M. *et al.* Understanding the Structural Basis for Substrate and Inhibitor Recognition in Eukaryotic GH11 Xylanases. *J. Mol. Biol.* **375,** 1293–1305 (2008).
